# The Biotinidase Gene Variants Registry: A Paradigm Public Database

**DOI:** 10.1534/g3.113.005835

**Published:** 2013-04-01

**Authors:** Melinda Procter, Barry Wolf, David K. Crockett, Rong Mao

**Affiliations:** * ARUP Institute for Clinical and Experimental Pathology, Salt Lake City, Utah 84108; † Departments of Medical Genetics and Pediatrics, Henry Ford Health System and the Center for Molecular Medicine and Genetics, Wayne State University School of Medicine, Detroit, Michigan 48202; ‡ Department of Pathology, University of Utah School of Medicine, Salt Lake City, Utah 84108

**Keywords:** biotinidase deficiency, biotinidase, *BTD* gene, mutation database, polymorphism

## Abstract

The *BTD* gene codes for production of biotinidase, the enzyme responsible for helping the body reuse and recycle the biotin found in foods. Biotinidase deficiency is an autosomal recessively inherited disorder resulting in the inability to recycle the vitamin biotin and affects approximately 1 in 60,000 newborns. If untreated, the depletion of intracellular biotin leads to impaired activities of the biotin-dependent carboxylases and can result in cutaneous and neurological abnormalities in individuals with the disorder. Mutations in the biotinidase gene (*BTD*) alter enzymatic function. To date, more than 165 mutations in *BTD* have been reported. Our group has developed a database that characterizes the known mutations and sequence variants in *BTD*. (http://arup.utah.edu/database/BTD/BTD_welcome.php). All sequence variants have been verified for their positions within the *BTD* gene and designated according to standard nomenclature suggested by Human Genome Variation Society (HGVS). In addition, we describe the change in the protein, indicate whether the variant is a known or likely mutation *vs.* a benign polymorphism, and include the reference that first described the alteration. We also indicate whether the alteration is known to be clinically pathological based on an observation of a known symptomatic individual or predicted to be pathological based on enzymatic activity or putative disruption of the protein structure. We incorporated the published phenotype to help establish genotype-phenotype correlations and facilitate this process for those performing mutation analysis and/or interpreting results. Other features of this database include disease information, relevant links about biotinidase deficiency, reference sequences, ability to query by various criteria, and the process for submitting novel variations. This database is free to the public and will be updated quarterly. This database is a paradigm for formulating databases for other inherited metabolic disorders.

Biotin is an essential water-soluble vitamin that is the coenzyme for four carboxylases in humans. The serum concentration of biotin depends on dietary biotin intake and recycling of endogenous biotin. Biotinidase is the enzyme that catalyzes the cleavage of biotin from biocytin or biotinylated peptides and releases biotin and lysine (Wolf *et al.* 1983a). Biotinidase deficiency (BD) (OMIM no. 609019) is an autosomal recessively inherited disorder caused by a defect in biotinidase resulting in multiple carboxylase deficiency (MCD, OMIM no. 253260). If untreated, BD can lead to neurologic and cutaneous symptoms (Wolf *et al.* 1983b; Wolf 2011; Pindolia *et al.* 2011). Profound BD is defined as having less than 10% of mean normal serum enzymatic activity, whereas partial BD is defined as having 10%–30% of mean normal serum enzymatic activity (Wolf 2003). Profound BD can result in seizures, hypotonia, developmental delay, respiratory problems, ataxia, hearing loss, optic atrophy, rash, and alopecia. Partial BD can result in any of the above symptoms, but it is usually milder or only manifests when the individual is stressed or suffers from an illness (Wolf *et al.* 1985). If a child develops hearing loss, optic atrophy, or developmental delay, the condition is usually irreversible (Wolf *et al.* 1983a). Essentially all states in the United States and many countries screen for this disorder at birth because early treatment with pharmacological doses of biotin can prevent the disorder from becoming symptomatic in children (Wolf 2002).

The human biotinidase gene is located on chromosome 3p25 and consists of four exons with a total length of 1629 base pairs. To date, there are more than 160 mutations of the *BTD* gene that have been reported. Four mutations, c.98_104delinsTCC, p.Q456H, p.R538C, and a complex mutation, p.D444H;A171T, are the mutations most frequently observed in individuals with BD and account for approximately 65% of affected individuals (Hymes *et al.* 2001). The p.D444H mutation alone is a “milder” mutation and in combination with a mutation for profound BD on the other allele results in partial BD.

Diagnosis of BD is usually confirmed by finding deficient biotinidase activity in serum (Cowan *et al.* 2010). Newborn screening has enabled identification of children with profound and partial BD. However, in some cases, enzymatic activity does not adequately differentiate partial deficiency from heterozygosity for profound deficiency; therefore, mutation analysis is necessary to confirm the diagnosis (Wolf 2010).

We have reported gene variant archives for eight diseases, such as galactosemia (*GALT*), multiple endocrine neoplasia type 2 (*RET*), juvenile polyposis syndrome (*SMAD4*), and X-linked Alport syndrome (*COL4A5*) (http://arup.utah.edu/). These clinically curated archives uniquely display genotype and associated phenotype information (Calderon *et al.* 2007; Crockett *et al.* 2010;Margraf *et al.* 2009; Wooderchak *et al.* 2010; Sumner *et al.* 2011). We now introduce a new disease database for BD and *BTD* gene variants (http://arup.utah.edu/database/BTD/BTD_welcome.php). This database was developed to serve as a reference for all sequence variations currently reported in the *BTD* gene as well as to provide the most up-to-date phenotypic information available. The database accepts new mutation submissions. To date, 165 variants of the *BTD* gene have been described (Hymes *et al.* 2001; Baykal *et al.* 2005; Couce *et al.* 2011; Funghini *et al.* 2002; Pomponio *et al.* 1997; Norrgard *et al.* 1999; Laszlo *et al.* 2003; Milankovics *et al.* 2007; Swango *et al.* 1998; Iqbal *et al.* 2010), of which 155 have been classified as pathogenic; one mutation is suspected of being pathogenic, and nine are suspected of being benign.

## Material and Methods

### *BTD* variant database objectives

The aim of the *BTD* variant database is to record all known variants in *BTD*, and their associated phenotypes and/or from abnormal newborn screening results. This database is freely available to the public, and users are encouraged to submit newly discovered variants to the database curator using the “Database Submission” Web page, regardless of publication status. The submission form can also be used to update clinical information or to clarify the classification or phenotype for existing sequence variants. The submission form requests information about the sequence variation; clinical findings, if any; publications; and the submitter’s contact information. Those submitting novel variants to the database are expected to follow their own Institutional Review Board (IRB) protocols or consenting processes according to their institution’s instructions. To maintain the accuracy and utility of the *BTD* database, content will be updated quarterly by using new sequence variation information from reports in the literature, database submissions, and updates; and routine clinical testing performed at ARUP Laboratories. The date of the latest update is displayed on the *BTD* “Home” Web page. ARUP’s *BTD* variant database is disease-specific, including clinical data when available. Each variant in the ARUP *BTD* database has been verified against the GenBank reference sequence accession numbers NC_000003.10, NM_000060.2, and NP_000051.1 and contains a “Comments” field including haplotype information where available. Previously unpublished submissions are updated to include references once data have been published.

### Data sources and limitations

The *BTD* database was constructed using gene sequence variation data published in the scientific literature since the *BTD* gene was first identified in individuals with BD, ascertained clinically or by newborn screening. To date, approximately 140 sequence variants have been found by searching PubMed (http://www.ncbi.nlm.nih.gov/sites/entrez) and Google (http://google.com/). Search terms included “*BTD*,” “biotinidase deficiency,” or “*BTD* mutation.” Occasionally, molecular sequencing results identify novel gene variants that are also included in this archive. The database was constructed using Human Genome Variation Society (HGVS) and Human Genome Organization (HUGO) Mutation Database Initiative recommendations for essential and optional content (http://www.hgvs.org/mdifaq.html). HGVS nomenclature recommendations for description of sequence variants were used to generate DNA and protein changes for this database. Entries were verified for position and name based on these sequences. All sequence variants in the database, including future updates, are designated based on their position in relation to the coding regions of the cDNA of *BTD* following recommendation listed in the HGVS (http://www.hgvs.org/mutnomen) and described in den Dunnen and Antonarakis (2000).

### Software

SQL tables (MySQL, Inc.) were organized to represent *BTD* mutation status, associated phenotype, literature references, and any known clinical information. PHP coding was performed in-house for dynamic HTML (hypertext markup language) display to render pages and display SQL tables. Graphics were generated using FusionCharts version 3 software (www.fusioncharts.com/). Web pages are hosted on a Mac OSX Apache server. *BTD* mutations were added to the database and edited using phpMyAdmin software (www.phpmyadmin.net).

## BTD Database Content

### Database Web site

**Figure 1:**
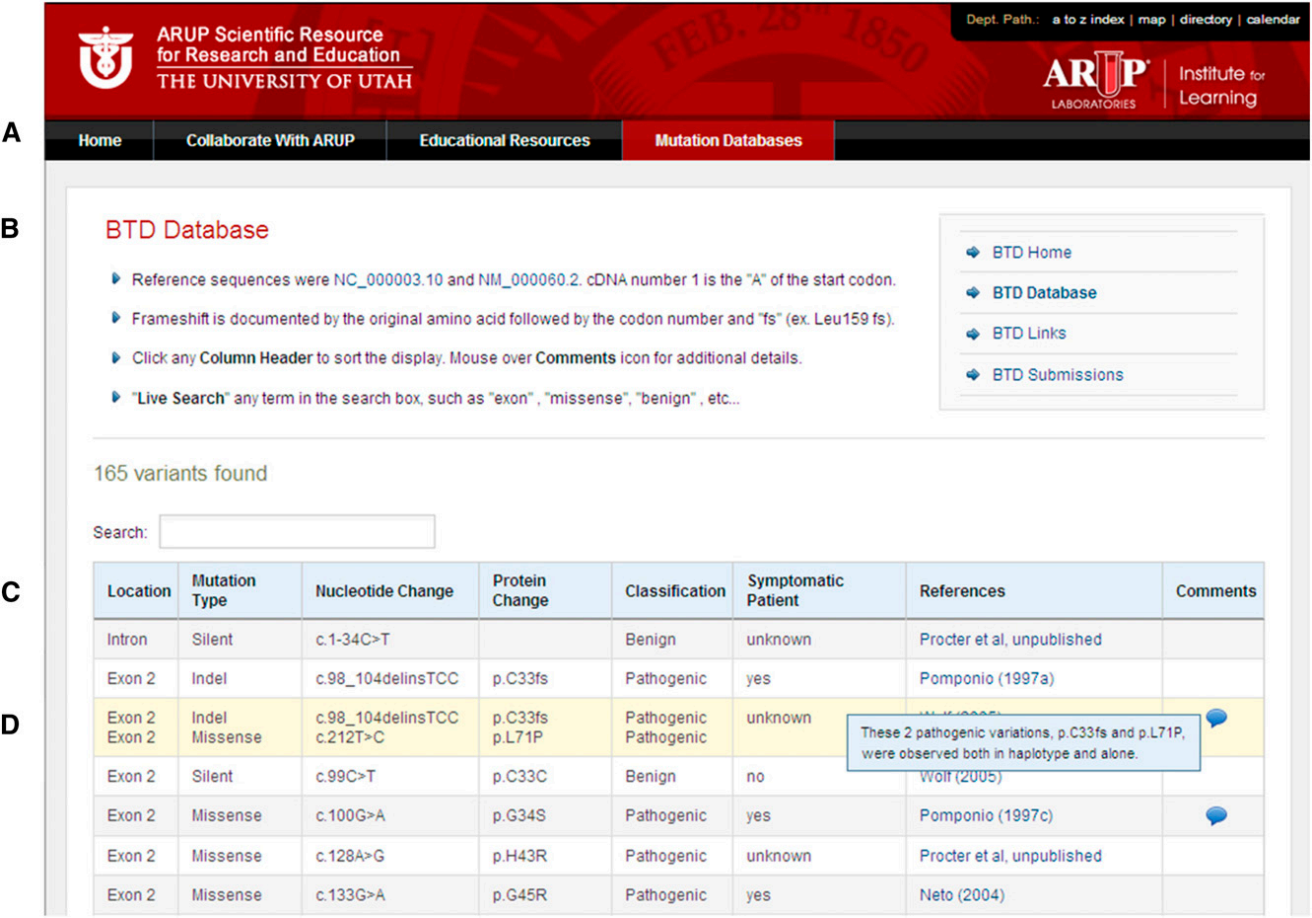
Biotinidase database display. (A) Tabs for navigation within the database Website for homepage, links display, search, submission and contact information. (B) Main database Website display. (C) Database column headings where gene variants can be arranged by clicking the sort arrows of select columns in either ascending or descending order. (D) Example of mouse-over function for “Comments” column following the literature reference.

The *BTD* database can be found under Disease Databases on the ARUP Online Scientific Resource Web page, http://www.arup.utah.edu/, or accessed directly by using the URL http://arup.utah.edu/database/BTD/BTD_welcome.php. Several Web pages can be navigated using the tabs shown in Figure 1A. The *BTD* “Home” Web page briefly describes profound and partial BD, the database goals, and gene sequencing tests available at ARUP Laboratories. The *BTD* “Links” Web page has hyperlinks to existing online resource for BD and *BTD*, including the National Library of Medicine’s Genetics Home Reference and OMIM and UniProt and the Human Protein Reference Database (HPRD) and *BTD* reference sequences used.

The database can be displayed in its entirety by accessing the “Display Database” Web page shown in Figure 1B. The database contained 165 entries as of January 2013. The default database is displayed according to the location of the sequence variation (5′ to 3′) within *BTD*. Figure 1 shows the database display columns Location, Mutation Type, Nucleotide Change, Protein Change, Classification, Phenotype, References, and Comments. Information that may further explain the data is displayed for each entry. The database also has search function capabilities such as location, classification, mutation type, literature reference, nucleotide change, or protein change. Searching may be performed from the *BTD* “Home” or “Search Database” pages.

The “Database Submissions” Web page can be used for a new submission of novel *BTD* sequence variation to the database or to update information for sequence variations already listed in the database. The “Contact Us” Web page has contact information, including E-mail addresses for the appropriate experts involved in developing and curating the database.

### Database display columns

#### BTD sequence variation location and mutation type:

**Figure 2:**
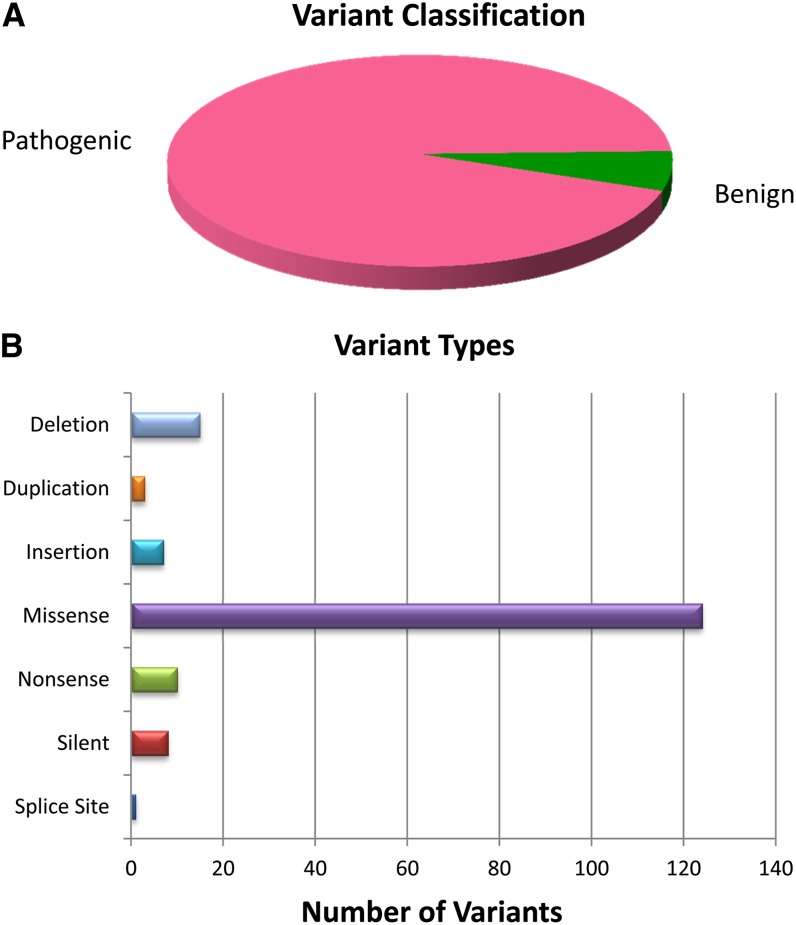
Summary of (A) variant classification and (B) types of mutations found in the *BTD* database.

The location column displays the exon (or intron) number for each *BTD* variant. Mutation type describes the deviation from the reference sequence leading to the change in the DNA. Common types of mutations include small deletions and insertions/duplications, missense, nonsense, splice site, and silent changes. Large deletions and duplications, larger than or equal to one exon, have not yet been reported in individuals with BD. *BTD* gene variant types are summarized in Figure 2.

#### BTD genotype:

The Nucleotide Change column displays the corresponding HGVS nomenclature. For cDNA numbering in the Nucleotide Change column of the *BTD* database, the first cDNA nucleotide (c.1) is the “A” of the ATG initiation codon.

#### BTD protein change:

For the Protein Change column, the single amino acid changes are listed in the database display. For splice-site variants, only the genotype is listed.

#### Classification of pathogenicity:

The classification definitions for pathogenicity are: Pathogenic, Suspected Pathogenic, Benign, Suspected Benign, or Uncertain. As is common practice, reduce or complete loss of biotinidase activity is assumed to be pathogenic and the author’s classification for each variant has used enzymatic activity, clinical phenotype and abnormal newborn screening results.

#### Symptomatic patients:

The status of patients’ clinical manifestations has been included where available. If no symptomatic patient had been observed due to early intervention, the symptomatic status has been stated as “unknown”. In the United States, symptomatic children are relatively rare due to the prevalence of newborn screening to diagnose infants early, as well as the ease of treatment to prevent symptoms.

#### References and comments:

The final two columns of the database feature links to literature resources and genotype/phenotype findings of each individual variant. As displayed in Figure 1, moving the cursor over comments are a key feature and unique part of this online resource. Comments for each *BTD* variant often contain additional information or evidence to support the classification designation. Importantly, clinical details, such as age or other medical conditions may be included, but protected information approximately the individual is never listed.

### Database contents

Currently, the database displays information and classification of 165 *BTD* variants. However, this number is actively increasing as the additional variants are described in the literature. In addition, pathogenic mutations that are detected during routine clinical testing of samples at ARUP Laboratories or those submitted by other laboratories will also be added to the database. As mentioned above, the database provides a linked reference to a PubMed abstract for each variant reported in the database. As the classification of each variant is directly based on evidence from the literature, the link to PubMed is a useful feature of this database. However, in cases where a variant has not yet been reported in the literature, the database will still provide the clinical and/or experimental or functional data indicative of its classification. Laboratories and database users may also contact curators via e-mail or telephone regarding database entries, additional references, or clarification of information posted on the site at any time.

Figure 2 summarizes the content of the *BTD* database by variant classification and mutation types. The database currently has information on 155 pathogenic mutations, one suspected pathogenic mutation, and nine suspected benign variants. A breakdown of the different types of mutations currently contained in the database is also shown in Figure 2B.

### Database access and features

The entire database content can be viewed using the display database option. Figure 1 shows an example of the Display Database option for all the sequence variant entries. Database entries may also be queried by certain criteria: variant location, classification (pathogenic, suspected pathogenic, benign, suspected benign and uncertain), mutation type (missense, nonsense, deletions, insertions or duplications, splice-site, or silent changes, nucleotide changes (based on cDNA position), and protein changes (based on codon number), or by the reference in the literature.

### Database sort and search functions

The database display can be sorted in ascending or descending order by clicking the arrows found on the Mutation Type, Classification, and References headings (Figure 1). In addition, users can search for variants by location, classification, mutation type, literature reference, nucleotide change, or protein change. The search box feature can be accessed from either the *BTD* “Home” or “Search Database” Web page. Upon selection of the search criteria, a drop-down list containing possible options appears that allows the user to further narrow search results. A free-text field is also provided which will allow the user to search for a specific nucleotide and/or protein (amino acid). These search features simplify accessibility to the database entries and ease of finding of a particular mutation.

### Clinical significance of the database

Clinical and research laboratories that offer full gene analysis for often encountered, rare variants must rely on the published literature or online resources to make informed decisions approximately the classification of the variant when reporting their findings. We developed this database to facilitate this process and assist those testing and interpreting results. The key feature of this database is the ease of access with which it provides pertinent gene variant information and associated genotype-phenotype observations. In addition, the database is updated and curated by individuals with expertise in both molecular genetics, as well as BD, ensuring that all of the information contained in the archive is up-to-date and evaluated with sufficient scientific rigor. Finally, as this database is a publicly available resource, with no login or membership requirements, it serves as an ideal centralized resource for all laboratories offering *BTD* gene analysis, as well as health professionals treating individuals with possible BD.
